# Geniposidic acid inhibits OVA-induced asthma by suppressing allergic airway inflammation and regulating gut microbiota

**DOI:** 10.3389/fimmu.2025.1549459

**Published:** 2025-02-25

**Authors:** Yang Zheng, Dengyu Gao, Hongyang Xie, Huafeng Geng

**Affiliations:** ^1^ Department of Gynecology, China-Japan Union Hospital of Jilin University, Changchun, Jilin, China; ^2^ Department of Anesthesiology, China-Japan Union Hospital of Jilin University, Changchun, Jilin, China

**Keywords:** asthma, geniposidic acid, gut microbiota, NF-κB, inflammation

## Abstract

Asthma is a serious chronic inflammatory disease of the respiratory system. In this study, we aimed to explore the role of geniposidic acid (GPA) in ovalbumin (OVA)-induced asthma in mice and to clarify its underlying mechanism. The mice were divided into control group, OVA group, OVA+GPA (12.5, 25, 50 mg/kg) groups. Inflammatory mediators were measured by ELISA. Gut microbiota was detected by 16S RNA sequencing. The results demonstrated that GPA attenuated OVA-induced lung injury, inflammatory cell infiltration, and mucus hypersecretion. OVA-induced IL-4, IL-5, IL-13, and IgE production was also inhibited by GPA. IFN-γ production was increased by GPA. Furthermore, GPA inhibited OVA-induced NF-κB activation and increased Nrf2 expression. In addition, GPA alleviated the dysbiosis of gut microbiota induced by OVA. After GPA treatment, the diversity and abundance of intestinal microbiota in asthma mice increased. At the phylum level, GPA significantly reduced the relative abundance of *Ligilactobacillus*, *Lachnospiraceae*, *Helicobacter*, and *Bacteroidales* and significantly increased the relative abundance of *Muribaculaceae* and *Muribaculum*. In conclusion, GPA protect mice against OVA-induced asthma through suppressing inflammation and regulating gut microbiota.

## Introduction

Allergic asthma is a chronic airway inflammatory response that involves various types of cells, cytokines, chemokines, and transcription factors (1). It is mainly characterized by extensive infiltration of inflammatory cells, release of inflammatory cytokines, high secretion of mucus, bronchospasm, and airway hyperresponsiveness (AHR) (2). At present, about 300 million people worldwide suffer from asthma, and its incidence rate and mortality have continued to rise in the past 20 years (3). However, there are currently no specific drugs for treating asthma. Corticosteroids, antihistamines, and leukotriene receptor antagonists are commonly used as asthma treatment drugs in clinical practice, but long-term use has poor efficacy and significant adverse reactions (4). The study of gut microbiota has become a hot topic in recent years, and gut microbiota is regarded as another “hidden organ” of the human body, closely related to metabolic diseases, digestive diseases, cardiovascular and cerebrovascular diseases, etc (5, 6). In recent years, with the continuous deepening of research, it has been found that intestinal microbiota imbalance is a high-risk factor for asthma (7, 8). Studies showed that gut microbiota can be used as a target for the treatment of asthma (9, 10).

NRF2 is an important transcription factor widely present in cells that regulates antioxidant enzymes and phase II detoxifying enzymes (11, 12). Under the stimulation of reactive oxygen species (ROS) or free electrons, NRF2 binds to antioxidant response elements (ARE) in the human nucleus, promoting the activation of downstream target genes such as antioxidant, anti-inflammatory proteins, and detoxifying enzymes (13). The structure of the lungs is complex and the pathogenesis is intricate. Recent research has found that in addition to immune response, oxidative antioxidant imbalance is also a key pathogenesis (14). In recent years, research has found that the Nrf2 signaling pathway is closely related to the occurrence and development of refractory respiratory diseases such as pulmonary fibrosis, asthma, lung cancer, and chronic obstructive pulmonary disease (15, 16). This pathway may serve as a target for the treatment of such diseases. A previous study found that anatabine can activate the Nrf2/HO-1 signaling pathway in lung tissue, inhibit oxidative stress in lung tissue of mice with bronchial asthma remission, improve mitochondrial function, and alleviate chronic airway inflammation (17).

Geniposidic acid (GPA), an iridoid glucoside isolated from Eucommia leaf, exhibits anti-inflammatory role (18). GPA exhibited neuroprotective role against Alzheimer’s disease in mice through inhibiting inflammation and apoptosis (19). GPA has been known to inhibit memory deficits and inflammation in APP/PS1 mice (20). GPA also alleviated lung inflammation and epithelial cell injury induced by LPS in rats (21). Furthermore, GPA could protect mice against colitis induced by DSS through attenuating inflammation and regulating gut microbiota (22). A previous study demonstrated that GPA could attenuate liver inflammation in cholestatic liver injury (23). However, whether GPA had protective role against asthma has not been studied. The purpose of this study was to investigate the protective role of asthma in mice.

## Materials and methods

### Materials and reagents

Geniposidic acid (purity>98%) was purchased from the China Institute for the Control of Pharmaceutical and Biological Products (Beijing, China). Aluminum hydroxide powder (#1.01091), OVA (#S7951) were purchased from Sigma (CA, USA). IL-4 (#SM4000B), IL-5 (#SM5000), IL-13 (#DY413-05), and IFN-γ (#SMIF00) ELISA kits were obtained from R&D Systems (Abingdon, UK). Antibodies for HO-1 (#70081), Nrf2 (#20733), p-NF-κB p65 (#3033), NF-κB p65 (#6956), p-IκBα (#2859), β-actin (#4967), and Lamin B (#13435) were purchased from CST (Danvers, MA).

### Animals and treatment

Sixty female BALB/c mice (aged 7–8 weeks) were purchased from Jilin University. All mice were acclimatized for a week before the experiment began. Mice are kept at 18-22 ℃, with good air circulation and free access to food and water. All animal procedures were performed and approved by the Animal Ethics Committee of Jilin University. The mice were divided into control, OVA, and OVA+GPA (12.5, 25, 50 mg/kg) groups. Except for the control group, other mice were used to establish asthma models using OVA combined with aluminum hydroxide stimulation (24, 25). On days 1, 7, and 14, 200 μL of sensitization solution (10 μg of OVA, 1 mg of aluminum hydroxide adjuvant, and physiological saline) were injected intraperitoneally. The control group mice were given an equal amount of physiological saline. On days 21-23, all mice were placed in an ultrasonic nebulizer and given 1% OVA in physiological saline at a total volume of 0.2 mL for 20 minutes of daily inhalation. The control mouse group was given physiological saline without OVA. GPA (12.5, 25, 50 mg/kg) was given 1 h before OVA treatment for the last three days.

### BALF collection and inflammatory cell count

After 24 hours of the last challenge, the mice were euthanized, fixed on an anatomical table, underwent tracheotomy. The BALF was collected by infused with 1 ml 0.9% sodium chloride solution after repeated aspiration for 3 times, and store it in a refrigerator at 4 °C. The total number of inflammatory cells in BALF were calculated using a blood cell counter, and then centrifuged at 3000 r/min for five minutes. The precipitated cell smear was stained with Diff Quick for classification and counting of inflammatory cells.

### Histopathologic evaluation

The left lung tissues of mice were fixed in a 4% paraformaldehyde solution overnight. Then, the lung tissue was subjected to routine paraffin embedding and sectioning steps such as dehydration, transparency, wax immersion, embedding, and sectioning. H&E staining and PAS staining were performed, and the morphological changes of the lung tissue in each group of mice were observed under an optical microscope.

### ELISA

The blood was collected and centrifuged at 4 °C at 3000rpm for 10 minutes to obtain the serum. The production of IL-4, IL-5, IL-13, and IFN-γ, in the BALF and IgE in serum were measured by the ELISA kits according to the manufacturer’s protocol.

### Western blot analysis

After the right lung tissue is fully lysed, it was centrifugated at 12 000 r/min at 4 °C for 10 min, and the supernatants were collected to measure the protein concentration. 30 μg protein sample was separated on 10% PAGE-SDS and stop electrophoresis when bromophenol blue runs to 1 cm from the bottom of the gel. The protein was transferred to PVDF membranes and soaked in 5% skimmed milk powder for 2 h at room temperature. Then, the membrane was incubated with primary antibodies overnight at 4 °C and wash the membrane with TBST for 8 minutes three times. The membrane was further incubated with HRP labeled goat anti-rabbit secondary antibody (1:5000) at room temperature for 1 hour, and washed the membrane with TBST for 8 minutes for three times. Finally, chemiluminescence development was performed, and the grayscale values of protein bands were detected using Image J software.

### Sequencing of the 16S ribosomal RNA genes of the gut microbiota

Thirty-six fecal samples (twelve in each group) were collected and send to Shanghai Meiji Biomedical Technology Co., Ltd. for 16S rDNA sequencing. Refer to the methods of previous study for DNA extraction, PCR amplification, and sequencing. Using Flash ver 1.2.3 software to process Paired end Illumina MiSeq sequences. The clustering analysis of operational taxonomic units (OTUs) with significant numbers at a similarity level of 97% was performed using Usearch ver 5.2.236. Using Rver 3.2 and Vegan software to analyze the variance statistics (ANOVA), non-metric multidimensional scaling (NMDS), and linear discriminant analysis (LDA) of fungal communities. Use PICRUSt1 software to predict the functional information of endophytic bacterial communities.

### Statistical analysis

This experiment used SPSS 19.0 software to analyze the data of each group, and the measurement data was expressed as ± S.E.M. Multiple group comparisons were conducted using one-way analysis of variance, and pairwise comparisons between groups were conducted using LSD-t-test. The difference was statistically significant when P<0.05. 16S rRNA sequencing data for all samples have been deposited in NCBI and are publicly available as of the date of publication (PRJNA1209398).

## Results

### GPA attenuates OVA-induced lung histopathological changes

**Figure 1:**
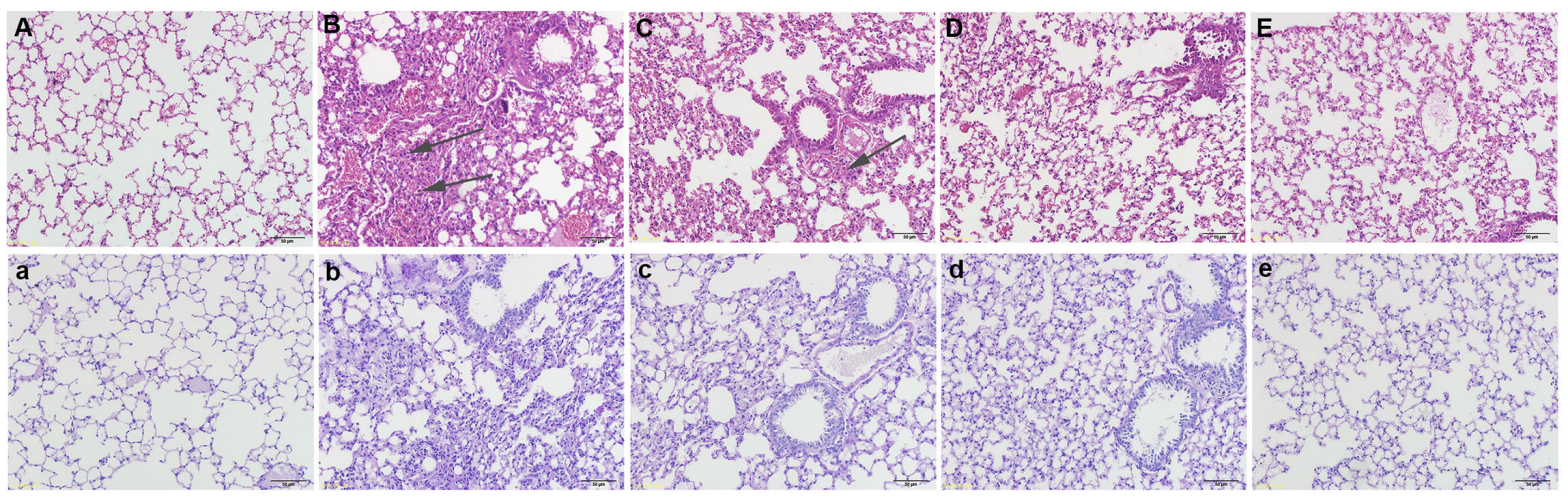
GPA attenuates OVA-induced lung histopathological changes (magnification 200×). (H&E staining) **(A)** Control group, **(B)** OVA groups, **(C-E)** OVA+ GPA (12.5, 25, 50 mg/kg) groups. (PAS staining) a: Control group, b: OVA groups, c-e: OVA+ EC (12.5, 25, 50 mg/kg) groups.

The H&E staining results showed that compared with the control group, the bronchial mucosa in the OVA group was damaged (**Figure 1A**), with a large number of inflammatory cells infiltrating the submucosa, and the airway wall thickened significantly (**Figure 1B**). GPA treatments significantly reduced the infiltration of inflammatory cells (**Figures 1C–E**). The PAS staining results showed that in the OVA group, there was a significant increase in goblet cells and mucus secretion around the airway (**Figure 1B**). GPA treatments effectively reduced the positive cell count and inhibited mucus hypersecretion (**Figures 1C–E**). These data revealed that GPA had protective role against OVA-induced asthma.

### Effects of GPA on OVA-induced inflammatory cell numbers in BALF

**Figure 2:**
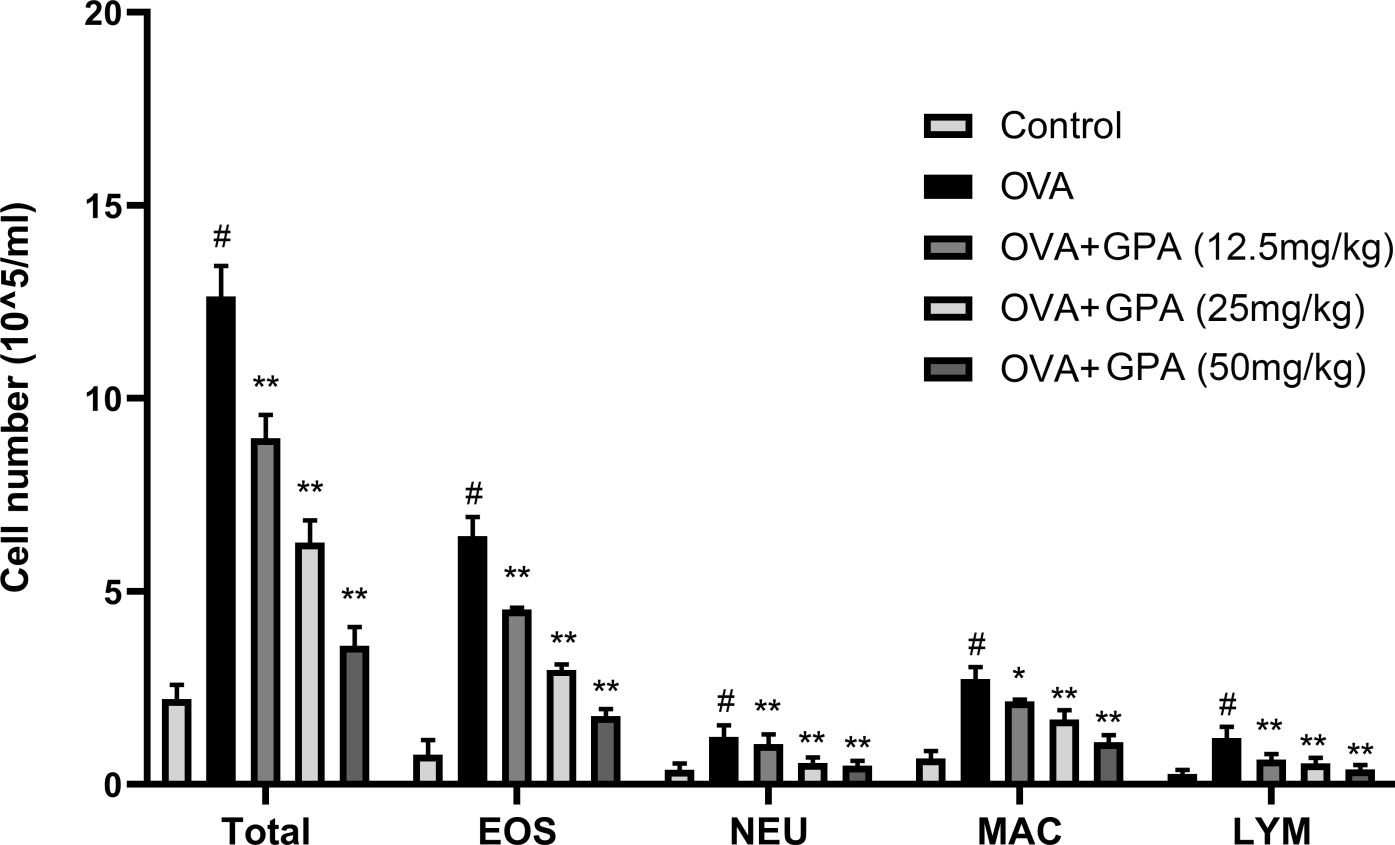
GPA inhibits inflammatory cell numbers in BALF. The data of this study are presented as mean ± SD of three parallel measurements. p#<0.01 vs. control group, p*<0.05 vs. OVA group. p**<0.01 vs. OVA group.

Statistical analysis of the number and classification of inflammatory cells in each group’s BALF revealed a significant increase in the total number of inflammatory cells in the OVA group compared to the control group (P<0.05), with eosinophils being the most prominent. Compared with the OVA group, the growth of eosinophils, lymphocytes, and macrophages in the OVA+GPA group was markedly inhibited (P<0.01) (**Figure 2**).

### Effects of GPA on OVA-specific IgE production in serum

**Figure 3:**
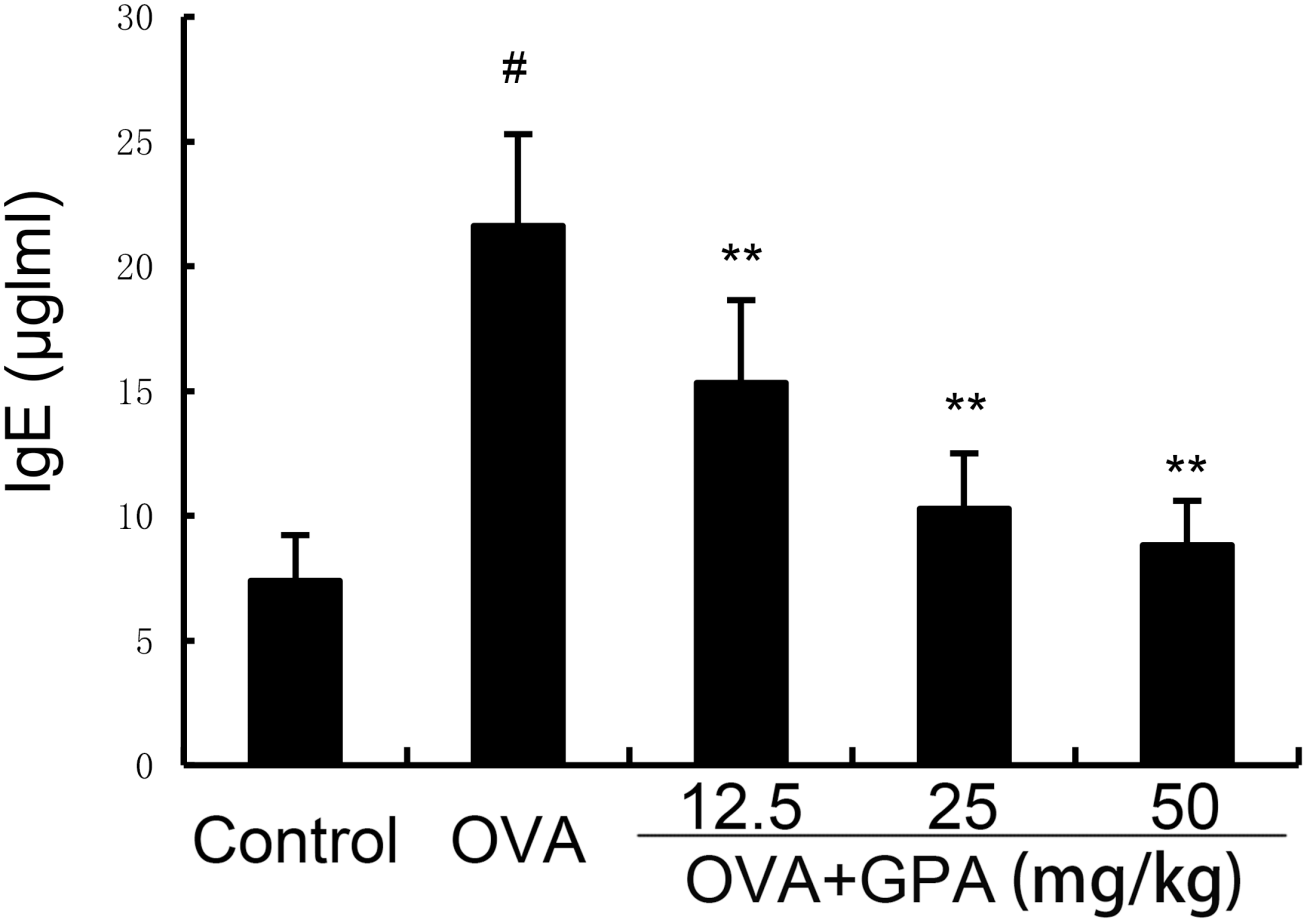
GPA attenuates OVA-specific IgE production. The data of this study are presented as mean ± SD of three parallel measurements. p#<0.01 vs. control group, p**<0.01 vs. OVA group.

As demonstrated in **Figure 3**, compared with the control group, the model group mice showed a significant increase in IgE production (P<0.01). GPA treatment obviously decreased IgE production induced by OVA (P<0.05 or P<0.01).

### Effects of GPA on OVA-induced IL-4, IL-5, IL-13, and IFN-γ production

**Figure 4:**
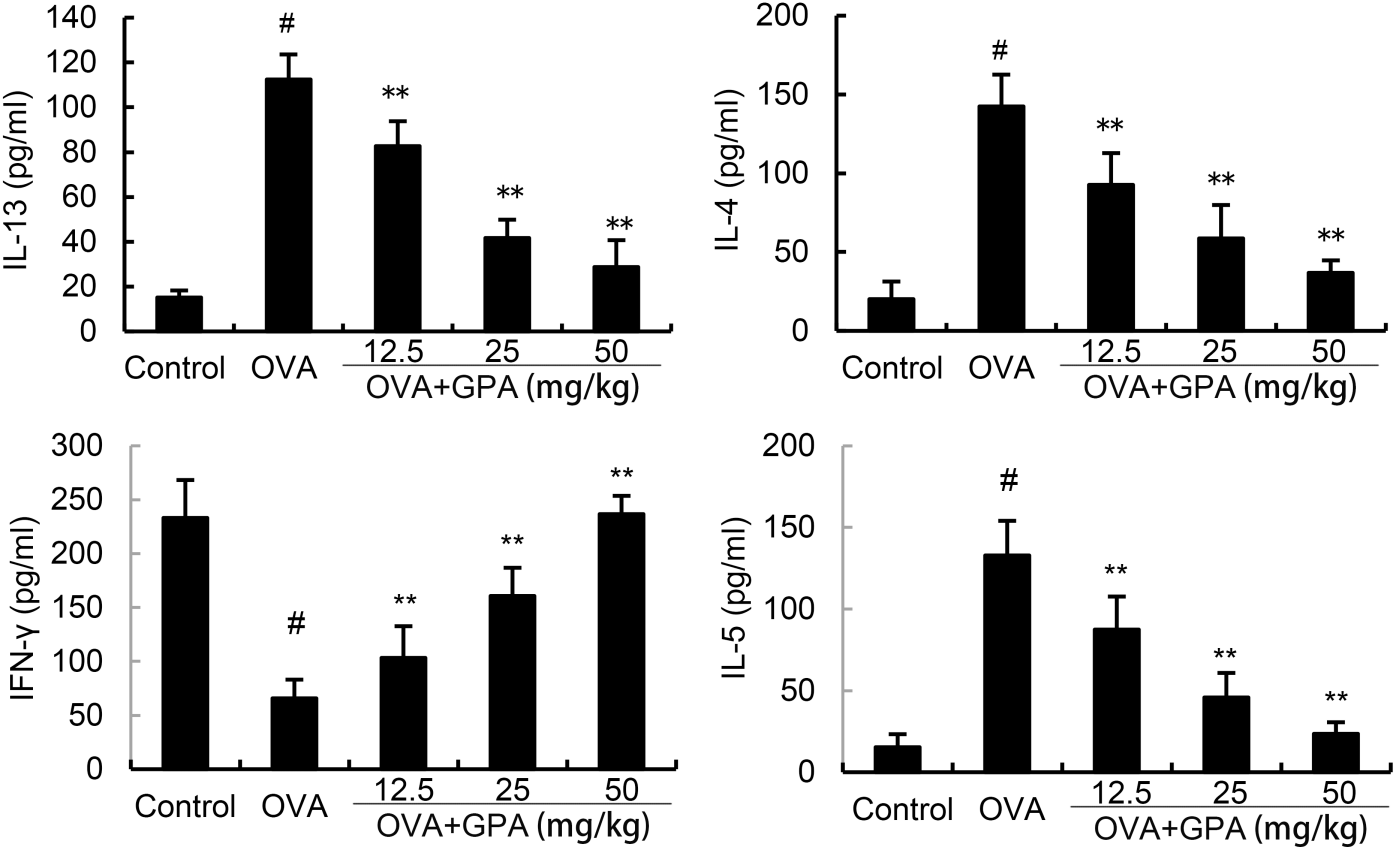
Effects of GPA on OVA-induced cytokine production. The data of this study are presented as mean ± SD of three parallel measurements. p#<0.01 vs. control group, p**<0.01 vs. OVA group.

The levels of Th1 and Th2 related cytokines in BALF of allergic asthma mice were detected by ELISA. The results are shown in **Figure 4**, compared with the control group, the model group mice showed a significant increase in IL-4, IL-5, IL-13 (P<0.01) and a significant decrease in IFN-γ level in BALF (P<0.01). GPA treatment obviously decreased IL-4, IL-5, IL-13 production and increased IFN-γ production (P<0.05 or P<0.01).

### Effects of GPA on OVA-induced NF-κB activation in lung tissue

**Figure 5:**
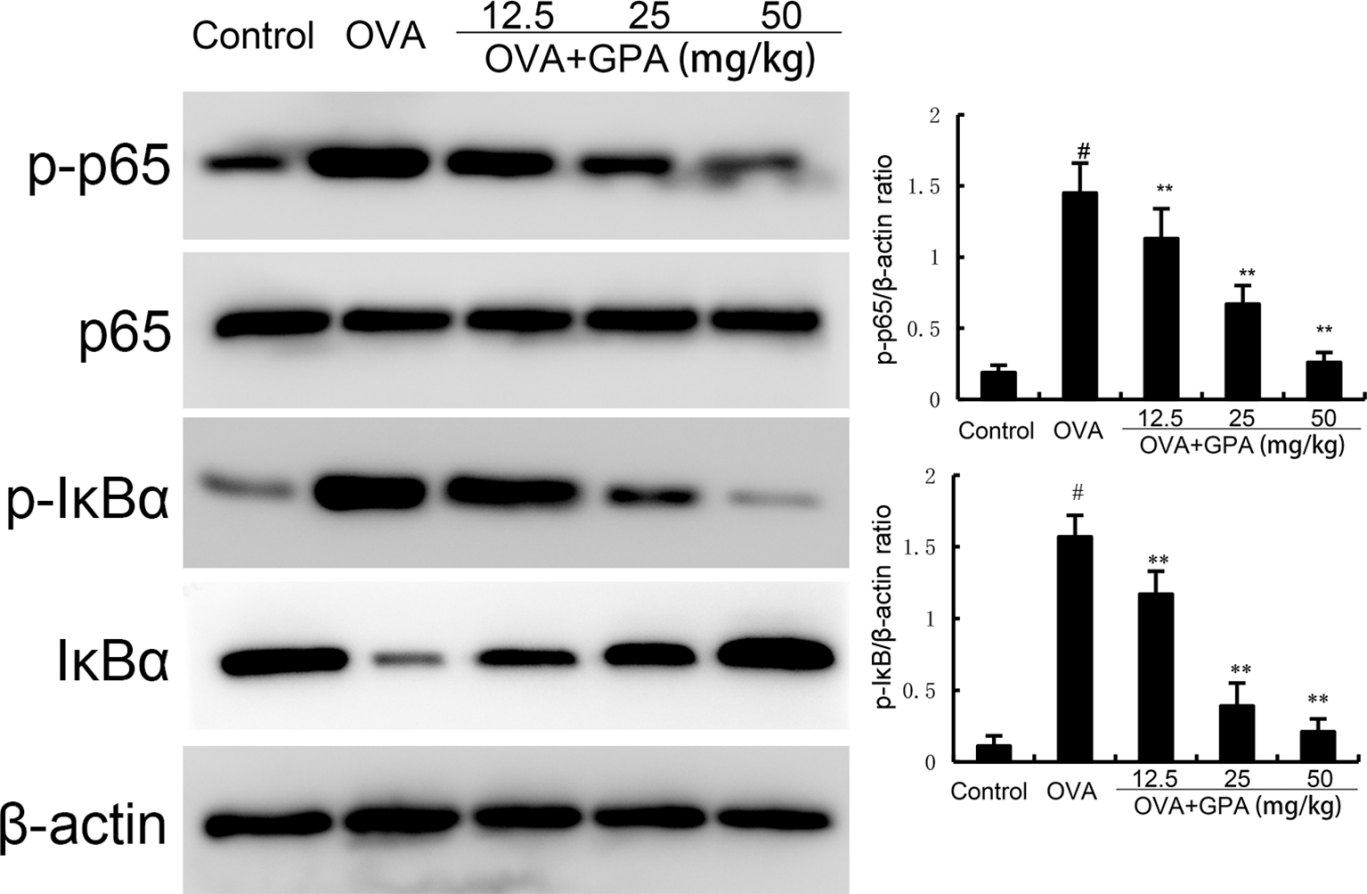
The role of GPA on OVA-induced NF-κB activation. The data of this study are presented as mean ± SD of three parallel measurements. p#<0.01 vs. control group, p**<0.01 vs. OVA group.

Compared with the normal group, the protein expression of p-NF-κB p65 and p-IκBα in the lung tissue of OVA group mice increased (P<0.05). Compared with the OVA group, the protein expression of p-NF-κB p65 and p-IκBα in the GPA group decreased (P<0.05), as shown in **Figure 5**.

### Effects of GPA on Nrf2 and HO-1 protein expression in lung tissue

**Figure 6:**
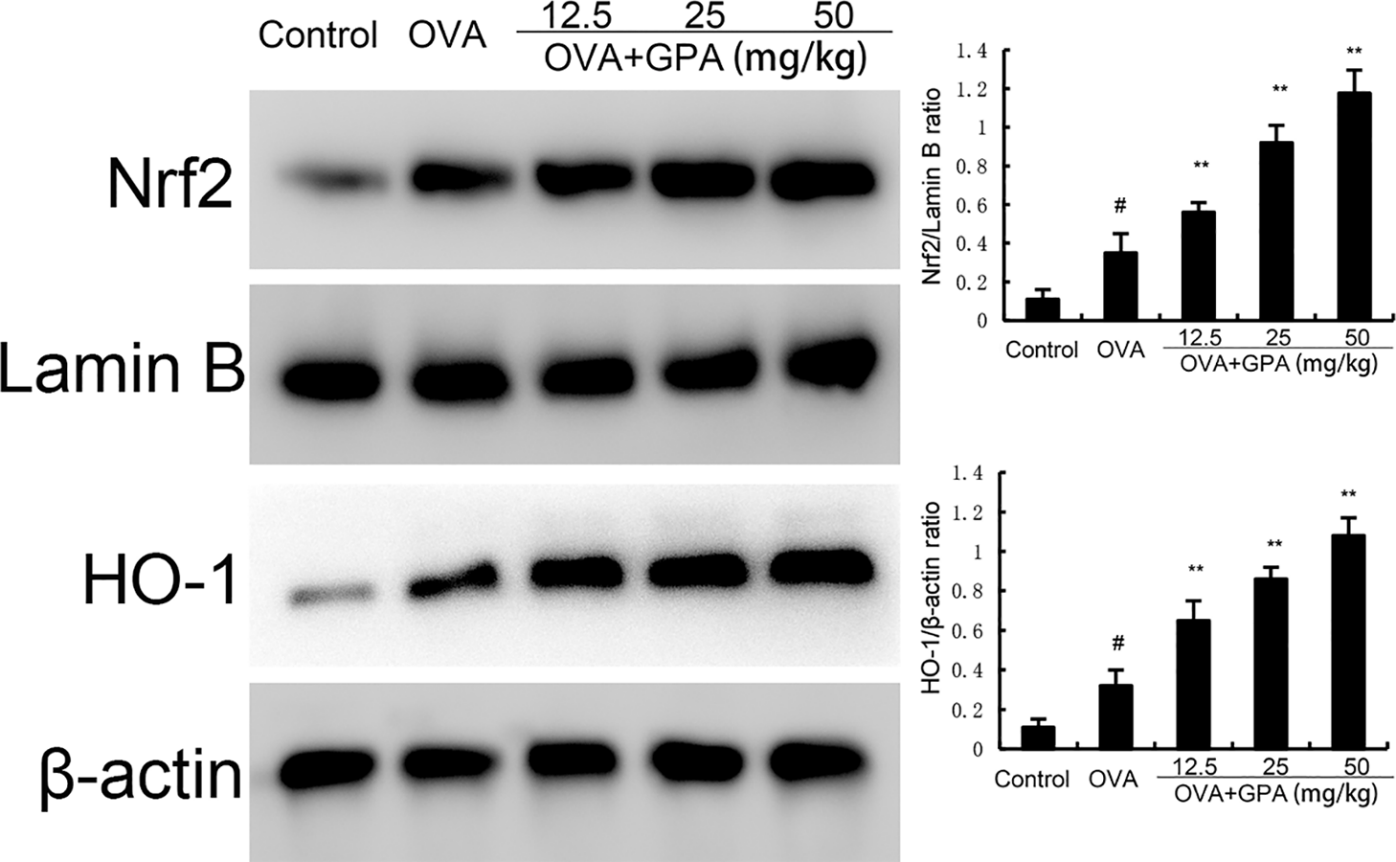
Effects of GPA on Nrf2 and HO-1 protein expression. The data of this study are presented as mean ± SD of three parallel measurements. p#<0.01 vs. control group, p**<0.01 vs. OVA group.

Compared with the normal group, the protein expression of Nrf2 and HO-1 in the lung tissue of OVA group mice increased (P<0.05). Compared with the OVA group, the protein expression of Nrf2 and HO-1 in the GPA group increased markedly (P<0.05), as shown in **Figure 6**.

### GPA alters gut microbiota diversity in OVA-treated mice

**Figure 7:**
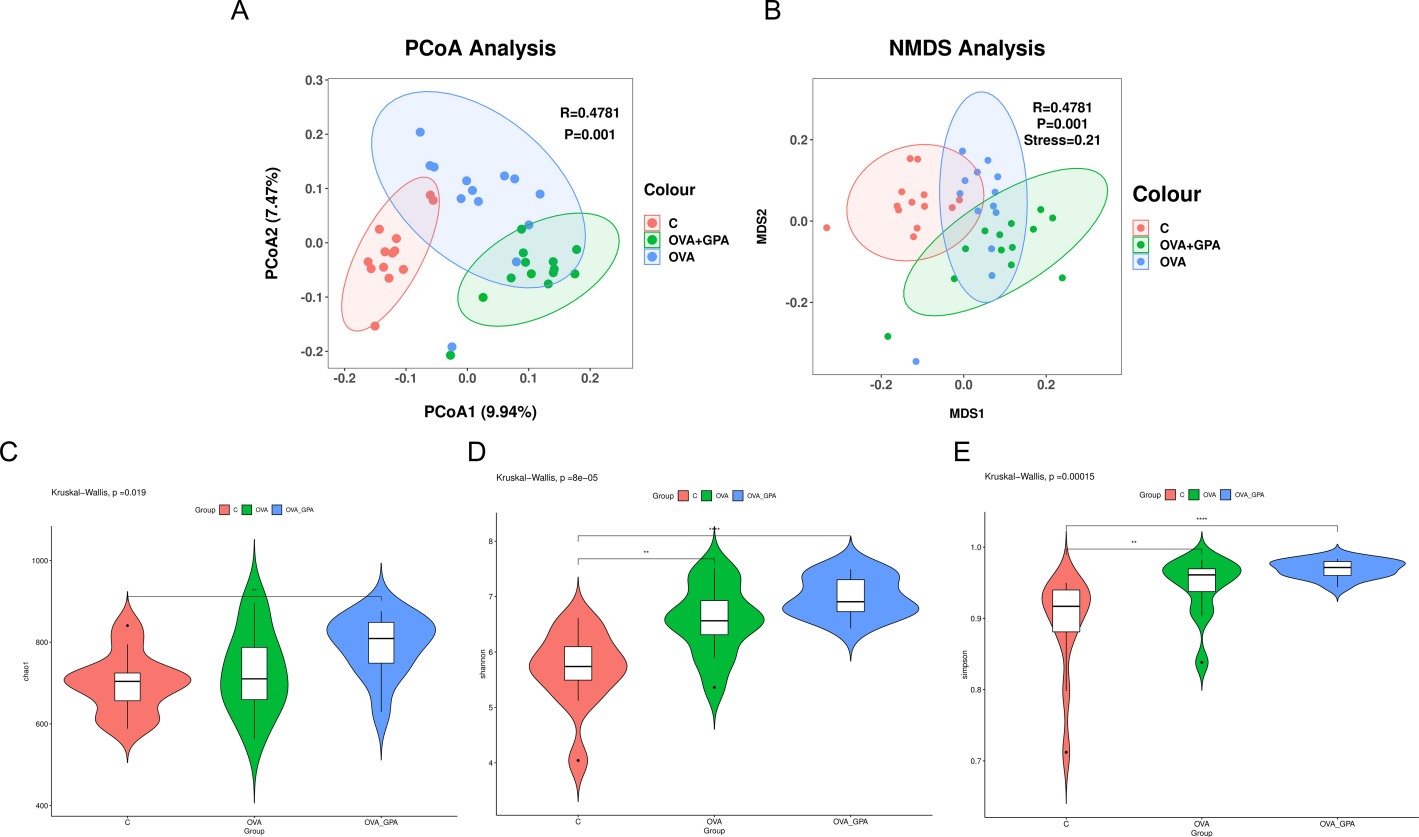
Alpha diversity and β-diversity analysis of the intestinal microflora for **(A, B)** the Chao1 index analysis **(C)**, the Shannon index analysis **(D)** and the Simpson index analysis **(E)**. Data are presented as the mean ± SD (n = 12). **P<0.05 in comparison with different groups, ****P<0.01 in comparison with different groups.

The Alpha diversity index reflects community richness and diversity, and the data showed that the Simpson, Chao, and Shannon indices of GPA treated mice were significantly increased (**Figure 7**), indicating that GPA could increase the richness and diversity of gut microbiota. The principal coordinate analysis (PCoA, **Figure 7** shows that the distances between the three groups are relatively far. This indicates that there are significant differences in the composition of gut microbiota between the blank group and the GPA group compared to the model group. OVA can significantly alter the species composition of gut microbiota in normal mice, and GPA can regulate OVA induced gut microbiota imbalance, bringing the species composition of gut microbiota closer to normal gut microbiota.

### GPA alters the composition of the gut microbiota in OVA-treated mice

**Figure 8:**
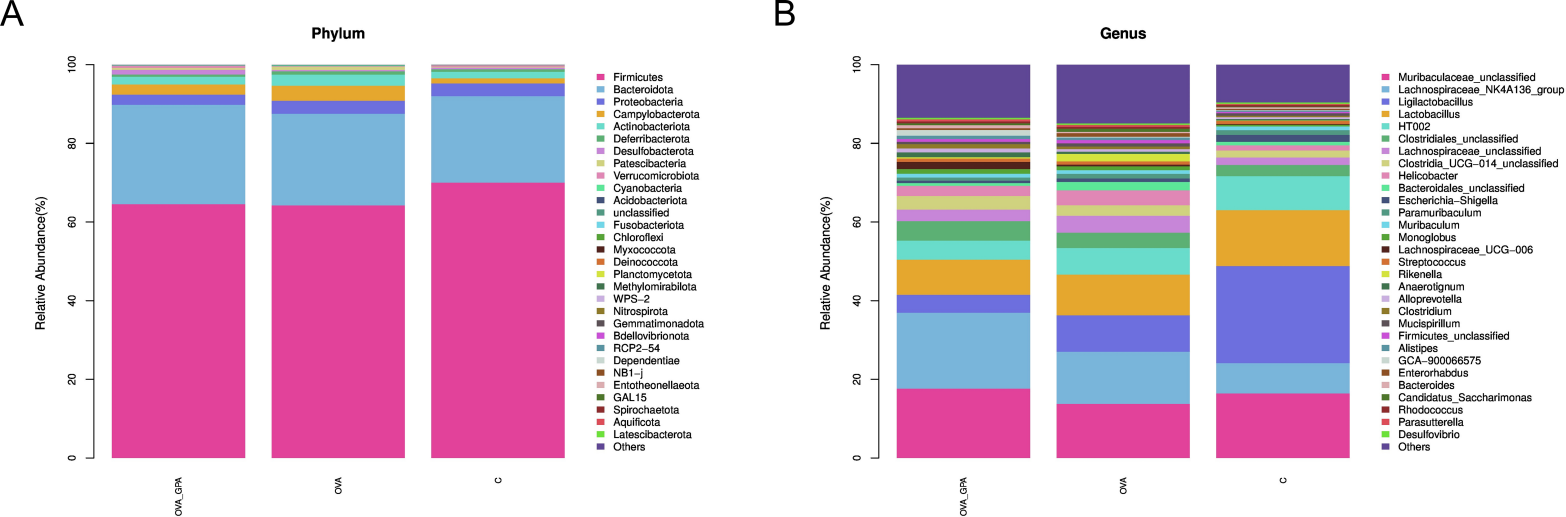
**(A)** Gut microbiota compositions of different groups at phylum levels. **(B)** Gut microbiota compositions of different groups at genus levels.

At the phylum level (**Figure 8A**), compared with the blank group, the OVA group showed a significant decrease in the relative abundance of Bacteroidota, Desulfobacterota, and Verrucomicrobiota, which the relative abundance of Proteobacteria, Campylobacteria, Actinobacteriota, Deferribacterota, and Patescibacteria increased significantly. Compared with the OVA group, the GPA group significantly reduced the relative abundance of Proteobacteria, Campylobacteria, Actinobacteriota, Deferribacterota, and Patescibacteria and significantly increased the relative abundance of Bacteroidota, Desulfobacterota, and Verrucomicrobiota. At the genus level (**Figure 8B**), compared with the blank group, the OVA group showed a significant decrease in the relative abundance of Muribaculaceae and Muribaculum, which the relative abundance of Ligilactobacillus, Lachnospiraceae, Helicobacter, and Bacteroidales increased significantly. Compared with the OVA group, the GPA group significantly reduced the relative abundance of Ligilactobacillus, Lachnospiraceae, Helicobacter, and Bacteroidales and significantly increased the relative abundance of Muribaculaceae and Muribaculum.

## Discussion

Asthma is a chronic inflammatory disease of the airway that involves multiple cells and cellular components, accompanied by recurrent symptoms such as wheezing, coughing, chest tightness, and shortness of breath (26). Epidemiological investigation shows that the incidence rate and mortality of asthma are increasing year by year, and there is a lack of specific drugs for the treatment of asthma in modern medicine, so there is an urgent need to develop alternative drugs that can effectively control or treat asthma (27). GPA has been reported to exhibit anti-inflammatory role. This experiment successfully established an allergic asthma model through OVA induction to study the intervention effect of GPA on allergic asthma. The results showed that GPA could protect mice against asthma via inhibiting inflammation and regulating gut microbiota.

When the body encounters external stimuli, Th cells are activated, forming a subgroup of effector cells, mainly including Th1 and Th2 cells (28). The balance of Th1 and Th2 cells plays a major role in the regulation of cellular and humoral immune responses (29). When the balance of Th1/Th2 cell differentiation is disrupted, the IFN-γ secreted by the Th1 subgroup and the IL-4, IL-5, and IL-13 secreted by the Th2 subgroup also exhibit an imbalance in the secretion of inflammatory cytokines (30). IL-4 and IL-5 regulate the maturation and release of eosinophils in the bone marrow, while IL-13 promotes B cell differentiation and plays a leading role in airway inflammation, airway hyperresponsiveness, and mucus secretion (31). These inflammatory cytokines are key factors in the pathogenesis of asthma (32). It is also a direct effector that leads to sustained inflammatory response and airway remodeling. After GPA treatment, the number of inflammatory cells and levels of IL-4, IL-5, and IL-13 in BALF of mice decreased, while IFN-γ level increased. H&E and PAS staining showed a reduction in airway inflammation, mucus secretion, and collagen deposition in mice. These results indicate that GPA can effectively alleviate the progression of asthma airway inflammation. The Nrf2 pathway is closely related to oxidative stress, inflammatory response, and apoptosis (33, 34). A previous study demonstrated that Nrf2 deficiency could increase airway inflammation in mice of asthma (35). Meanwhile, a previous study showed that Vitamin E could protect mice against asthma through activating Nrf2 signaling pathway (36). Furthermore, edaravone could attenuate lung inflamamtion through increasing protein expresion of Nrf2 and HO-1 in asthma model (37). These studies indicated that activation of Nrf2 exhibited protective role against asthma. In this study, GPA significantly up-regulated the expression of Nrf2 and HO-1, indicating GPA attenuated asthma through Nrf2/HO-1 signaling pathway.

Recent studies have demonstrated that gut microbiota dysbiosis can contribute to asthma onset and exacerbation, prompting investigations into therapeutic strategies to correct this imbalance. Probiotics and prebiotics, known for their ability to modulate gut microbial compositions, were discussed as potential interventions to restore immune homeostasis.

Multiple studies have shown that gut microbiota plays an undeniable role in the formation and treatment of asthma, and the composition and metabolites of gut microbiota affect lung health to varying degrees (38). Disturbance of gut microbiota could lead to the onset and exacerbation of asthma. Disturbance of gut microbiota can lead to damage to the intestinal mucosal barrier, leading the translocation of bacterial components, such as lipopolysaccharides (LPS), into the bloodstream (39, 40). This can trigger a systemic inflammatory response and activate immune cells, which may then migrate to the lungs and exacerbate the asthmatic inflammation. Secondly, the dysbiosis induced changes in the immune system may disrupt the normal Th1/Th2 balance (41). Therefore, maintaining stable gut microbiota is crucial for preventing and treating asthma. Recently, numerous studies have found that many probiotics and prebiotics can prevent and treat asthma by regulating the gut microbiota (42, 43). In this study, the diversity of gut microbiota was reduced and after GPA intervention, both α diversity and β diversity were significantly improved. At the genus level, GPA significantly reduced the relative abundance of Ligilactobacillus, Lachnospiraceae, Helicobacter, and Bacteroidales and significantly increased the relative abundance of Muribaculaceae and Muribaculum. Muribaculaceae is considered a biomarker of healthy gut microbiome, which can encode enzymes required for the removal of O-glycan terminal sialic acid and sulfate residues on mucin, playing an important role in the degradation process of intestinal mucin (44). The reduction of mucin can lead to increased intestinal permeability and damage to intestinal barrier function. Our findings support the growing body of evidence highlighting the importance of gut microbiota in asthma.

In conclusion, the results showed that GPA had protective role against asthma. GPA protected mice against OVA-induced asthma through suppressing inflammation and regulating gut microbiota.

## Data Availability

The datasets presented in this study can be found in online repositories. The names of the repository/repositories and accession number(s) can be found in the article/supplementary material.
